# Antibacterial Properties of Mussel-Inspired Polydopamine Coatings Prepared by a Simple Two-Step Shaking-Assisted Method

**DOI:** 10.3389/fchem.2019.00631

**Published:** 2019-09-25

**Authors:** Pegah Kord Forooshani, Elizabeth Polega, Kevin Thomson, Md. Saleh Akram Bhuiyan, Rattapol Pinnaratip, Mikhail Trought, Chito Kendrick, Yuesheng Gao, Kathryn A. Perrine, Lei Pan, Bruce P. Lee

**Affiliations:** ^1^Department of Biomedical Engineering, Michigan Technological University, Houghton, MI, United States; ^2^Department of Chemistry, Michigan Technological University, Houghton, MI, United States; ^3^Department of Electrical Engineering, Michigan Technological University, Houghton, MI, United States; ^4^Department of Chemical Engineering, Michigan Technological University, Houghton, MI, United States

**Keywords:** catechol, polydopamine, coating, hydrogen peroxide, antibacterial, surgical mesh

## Abstract

A simple two-step, shaking-assisted polydopamine (PDA) coating technique was used to impart polypropylene (PP) mesh with antimicrobial properties. In this modified method, a relatively large concentration of dopamine (20 mg ml^−1^) was first used to create a stable PDA primer layer, while the second step utilized a significantly lower concentration of dopamine (2 mg ml^−1^) to promote the formation and deposition of large aggregates of PDA nanoparticles. Gentle shaking (70 rpm) was employed to increase the deposition of PDA nanoparticle aggregates and the formation of a thicker PDA coating with nano-scaled surface roughness (RMS = 110 nm and Ra = 82 nm). Cyclic voltammetry experiment confirmed that the PDA coating remained redox active, despite extensive oxidative cross-linking. When the PDA-coated mesh was hydrated in phosphate saline buffer (pH 7.4), it was activated to generate 200 μM hydrogen peroxide (H_2_O_2_) for over 48 h. The sustained release of low doses of H_2_O_2_ was antibacterial against both gram-positive (*Staphylococcus epidermidis*) and gram-negative (*Escherichia coli*) bacteria. PDA coating achieved 100% reduction (LRV ~3.15) when incubated against *E. coli* and 98.9% reduction (LRV ~1.97) against *S. epi* in 24 h.

## Introduction

Surgical meshes are widely used to reinforce defective and weakened soft tissues in various types of prolapses including abdominal wall repair such as hernia repair (Baylón et al., 2017) and pelvic organ prolapse treatment (Todros et al., 2016). Hernia repair is one of the most common surgical procedures with more than 20 million procedures per year worldwide (Kingsnorth, 2004). Pelvic organ prolapse is also reported in up to 50% of women above 50 and those who have had a history of vaginal childbirth (Maher et al., 2013). The most commonly used mesh materials are polypropylene (PP) due to their favorable mechanical and biological properties (Clavé et al., 2010; Wolf et al., 2014). PP meshes have demonstrated excellent durability and biocompatibility (i.e., biologically inert and non-carcinogenic). However, infections associated with surgical mesh are one of the most common post-surgical complications, which result in patient morbidity, prolonged hospitalization, hernia recurrence, the necessity to remove the contaminated mesh, and the need for a follow-on surgical repair (Waldvogel and Bisno, 2000; Narkhede et al., 2015). To prevent microbial infection and biofilm formation, antibacterial coatings appear to be an effective and inexpensive approach. However, it is often difficult to create a stable coating on PP surface due to its inert and non-polar nature (Salimi, 2012; Chashmejahanbin et al., 2014).

Marine mussels secrete protein-based adhesives, which enable them to anchor to various surfaces in their wet and saline habitat (Lee et al., 2011; Kord Forooshani and Lee, 2017). These adhesives contain a unique catecholic amino acid, 3,4-dihydroxyphenylalanine (DOPA) (Danner et al., 2012; Wei et al., 2012). The catechol side chain of DOPA is responsible for strong interfacial binding and rapid solidification of the adhesive proteins. Catechol forms strong reversible non-covalent and irreversible covalent interactions with both organic and inorganic surfaces, which include hydrogen bonding, π-π electron interaction with another aromatic ring (Waite, 1987), π-cation interaction with a positively charged cationic group (Gallivan and Dougherty, 2000), reversible complexation with metal ions (Waite, 1987), co-ordination bonds with metal oxide surfaces (Kummert and Stumm, 1980; Lee et al., 2006), and covalent cross-linking with nucleophilic functional groups (Sugumaran et al., 1989; Waite, 1990). Additionally, catechol autoxidizes in the presence of molecular oxygen (O_2_) by one- and two-electron oxidation to form semiquinone and quinone, respectively (Waite, 1987; McDowell et al., 1999). During the oxidation process, reactive oxygen species (ROS) such as superoxide anions (${\text{O}}_{2}^{\cdot -}$) and hydrogen peroxide (H_2_O_2_) are generated as by-products (Mochizuki et al., 2002; Meng et al., 2015; Forooshani et al., 2017). Previously, we prepared catechol-functionalized microgels, which generated up to 4 mM of H_2_O_2_ when the microgels were simply submerged in an aqueous solution with mildly basic pH (pH ≥ 6) (Meng et al., 2019). The generated H_2_O_2_ was antimicrobial against both gram-positive (*Staphylococcus epidermidis*) and gram-negative (*Escherichia coli*) bacteria and antiviral against both non-enveloped porcine parvovirus and enveloped bovine viral diarrhea virus.

Here, we seek to functionalize PP mesh with H_2_O_2_-generating catechol moieties using a simple polydopamine (PDA) coating technique. During the oxidation-induced polymerization, PDA is deposited as an adhesive coating onto a surface (Lee et al., 2007, 2019). PDA can directly coat various types of surfaces ranging from metal to inert polymers and ceramic regardless of the shape and size of the substrate (Lee et al., 2007; Ren et al., 2011; Black et al., 2013). The resulting PDA coating is extremely thin (0.1–1 × 10^−9^ m) and will not significantly affect the geometry and structure of the functionalized substrates (Jiang et al., 2011). PDA coating remains chemically reactive for further functionalization with nucleophile-containing polymers or metal ions to create multifunctional surface coatings (Lee et al., 2019). However, during the coating process, dopamine undergoes extensive oxidative cross-linking to form structures that resemble natural melanin (Lim et al., 2016). This may decrease the content of the reduced form of catechol that is needed for the generation of H_2_O_2_.

**Figure 1:**
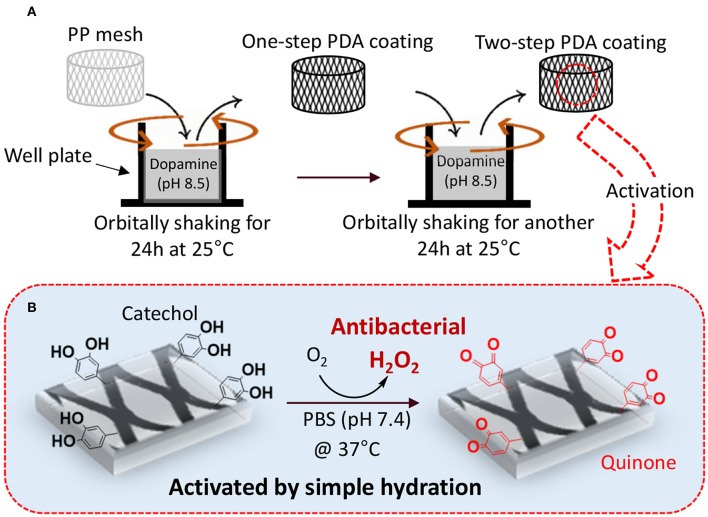
Schematic representation of two-step PDA coating with gentle shaking **(A)**. PP mesh is immersed in concentrated dopamine solution (pH 8.5) and orbitally shaken for 24 h at 25°C to prepare one-step coating. In the second step, PDA-coated mesh is further exposed to a freshly prepared dopamine solution (pH 8.5) with reduced concentration and incubating for another 24 h at 25°C. The PDA-coated mesh contains catechol moieties that can be activated to generate H_2_O_2_ by simply hydrating the mesh in a PBS with neutral pH **(B)**.

We proposed a two-step PDA coating process under gentle shaking to create PDA-coated PP meshes with antimicrobial properties (Figure 1). The first of the two steps was aimed at creating a stable base layer of PDA coating while the second step was aimed at anchoring dopamine to the existing PDA layer that can be oxidized to generate H_2_O_2_. Injecting the freshly prepared dopamine solution during the multi-step coating process can potentially avoid reaching a film growth plateau when dopamine polymerization reaches a steady state and can encourage additional film deposition (Bernsmann et al., 2009; Alfieri et al., 2018). PDA coating prepared under shaking [<200 rotations per minute (rpm)] has been previously shown to create nano-scaled surface roughness (Su et al., 2016), which can potentially increase the surface area of the coating for rapid oxidation and release of H_2_O_2_. Agitation of the dopamine solution potentially facilitates exchange of O_2_ with the atmosphere (Bernsmann et al., 2009), which has been proven to be key in promoting PDA coating formation (Bernsmann et al., 2011). The effect of multi-step coating process and shaking on the PDA coating was examined using physicochemical and morphological characterizations. The ability for the PDA-coated mesh to generate H_2_O_2_ and its antibacterial activity was determined.

## Materials and Methods

Dopamine hydrochloride and phosphate buffered saline (PBS, BioPerformance certified, pH 7.4) were purchased from Sigma. Tris base and 12 M hydrochloric (HCl) acid was purchased from Fisher Scientific. H_2_O_2_ (30% stock solution) was from Avantor (Center Valley, PA). Pierce Quantitative Peroxide Assay Kit with sorbitol and dihydroethidium (DHE) was purchased from Thermo Scientific (Rockford, IL). Mueller Hinton Agars (28 ml fill, 15 × 5 l nr) was purchased from Hardy Diagnostics (Santa Maria, CA). Disposable inoculating loop (10 μl, yellow, sterile) was purchased from VWR North American. Anprolene® gas sterilization and gas refills (ethylene oxide) were purchased from Andersen Sterilizers, Inc. *S. epi* (ATCC 12228) and *E. coli* (ATCC 11775) were purchased from the American Type Culture Collection (ATCC, Manassas, Virginia). The PP flow mesh (~1/3 mm thick with ~70% void space) was purchased from Diversified Biotech. PP sheets (24″ × 24 ″ × 1/16″, semi-clear white) were purchased from McMaster-Carr. Gold Seal™ Plain Microscope Slides was from Thermo Fisher Scientific. Fetal bovine serum (FBS), penicillin–streptomycin (10 U/ml), catalase from bovine liver (2,000–5,000 U/mg), and Calcein-AM/ethidium bromide for cell live/dead stain were purchased from Invitrogen, Thermo Fisher Scientific (Rockford, IL).

### Preparation of PDA Coatings

The PP meshes were cut into two separate pieces to fit vertically along the circumference of the wall (a rectangular piece, 70 × 7 mm) and the bottom (circular piece with radius = 9 mm) of a well in a 12-well tissue culture plate. The approximate surface area available for coating was 223 mm^2^. Three milliliters of freshly prepared dopamine solution (2–22 mg ml^−1^ in 25 mM Tris–HCl buffer, pH 8.5) was added to each well. The samples either were shaken using an orbital shaker (70 rpm) or were not shaken for 24 h at 25°C to make the one-step samples. The formulations are denoted as a number based on the concentration of dopamine followed by the letters S or NS for shaken and the non-shaken samples, respectively. For example, 2S and 2NS indicate samples that were coated with 2 mg ml^−1^ of dopamine under shaken and non-shaken conditions, respectively.

To coat PP meshes using the two-step coating approach, meshes were first incubated with the freshly prepared dopamine solution (10 or 20 mg ml^−1^ dopamine HCl in 25 mM Tris–HCl buffer, pH 8.5) for 24 h at 25°C with or without shaking. The dopamine solution was removed and replaced with a freshly prepared dopamine solution (2 mg ml^−1^ dopamine HCl in 25 mM Tris–HCl buffer, pH 8.5) and incubated for another 24 h in the same preparation conditions as the first step. The two-step samples were denoted as 10-2 or 20-2 based on the coating concentration of each step followed by the letters S and NS for shaken and the non-shaken samples, respectively. For example, 10-2S indicates a mesh coated with 10 mg ml^−1^ of dopamine in the first step, 2 mg ml^−1^ of dopamine in the second step, and both under shaken condition. To create model coatings for surface characterization, surfaces such as PP sheets, glass slides, and gold electrode were immobilized on the wall of a 50-ml beaker and coated with PDA using the above-described protocols. All the coated meshes were washed in an acidified water bath (pH 3.5) for 24 h and further rinsed with 70% ethanol and air dried before use.

### Determination of H_2_O_2_ Concentration

PDA-coated meshes were placed in 12-well tissue culture plates and 2 ml of PBS (pH 7.4) was added in each well. The meshes were incubated for up to 48 h at 37°C with gentle agitation on a shaking plate. H_2_O_2_ concentration was quantified using the Quantitative Peroxide Assay Kit [i.e., ferrous oxidation–xylenol orange (FOX) assay] by following previously published protocols (Clement et al., 2002). A standard curve was prepared using a series of solutions containing 0–1 mM of H_2_O_2_. Effects of catalase on the H_2_O_2_ generation were evaluated by adding 2 ml of PBS containing 20–50 U/ml of bovine liver catalase and incubated at 37°C. The experiment was performed in triplicate, and the results are reported as mean ± standard deviation (SD).

### Characterization of the PDA Coatings

#### Chemical Analysis

PDA-coated meshes were characterized using ATR-Fourier transform infrared (ATR-FTIR) spectroscopy (Perkin Elmer Spectrum One spectrometer) at a resolution of 1 cm^−1^ with a scan rate of 800 scans per minute. X-ray photoelectron spectroscopy (XPS) was performed using a PHI 5800 instrument on PDA-coated meshes. A Mg anode was used to collect the XPS data, which was charge corrected with respect to aliphatic carbon at 284.6 eV. The C1s, N1s, and O1s regions were collected using a pass energy of 23.5 eV. The result was further analyzed using 100% Gaussian peaks and a Shirley background subtraction in OriginPro.

#### Morphological and Topological Analysis

PDA-coated meshes were first coated with 5-nm-thick Pt/Pd coating and characterized with a field emission scanning electron microscope (FE-SEM; Hitachi S-4700). Topography and the surface roughness of the PDA-coated meshes and the control mesh without coating were evaluated using Asylum MFP-3D Origin instrument. The images were collected using tapping mode (AC mode) with scan areas of 10 × 10 and 6 × 6 μm^2^ (i.e., the scan parameter is 256 points and lines with a scan rate of 1 Hz). An aluminum-coated cantilever was used, which contains a silicon nitride tip and a force constant of 40 N/m. Third-order flattening was performed on all the images to remove any frequency noise or disorientation of the image during scanning. Root mean squared roughness (RMS) and average roughness (Ra) values were reported for the 10 × 10 μm^2^ images. At least three measurements were performed on each sample and the average result was reported as mean ± SD.

#### 3D Profilometer

PDA film thickness was measured using a Filmetrics 3D Profilometer on PDA-coated glass slide. Kapton tape was used to mask half of the glass slide before PDA deposition, which was removed later to obtain a step edge to measure the coating thickness.

#### Contact Angle

The contact angles were measured by the so-called sessile drop technique on PDA-coated PP sheets and control PP sheet without PDA coating. A small drop of DI water (diameter of ~2 mm) with a resistivity of over 18 mΩ·cm was gently placed on the samples fixed on a flat stage. A side-view CCD camera (FLIR, Japan) recorded the shape and profile of the drop with the help of a light source on the other side. The intensity of the light has been adjusted to achieve the optimal image quality. Then, the images were processed by a self-coded MATLAB program to obtain the contact angles. To guarantee the accuracy of the experiments, each test was repeated at least three times, and the average result was reported as mean ± SD.

#### Cyclic Voltammetry

Cyclic voltammetry (CV) was performed using a custom-designed gold electrode as the working electrode (WE) (Figure S1). Kapton tape was used to mask the electrode's pattern on the surface of a glass slide before gold deposition. Gold film (100 nm thick) was deposited on the surface of the glass slide, which was further coated with PDA. Platinum wire and Ag/AgCl-containing electrode were used as the counter electrode (CE) and the reference electrode (RE), respectively. PBS buffer (pH 7.4) was used as the electrolyte (20 ml). The first cycle started from zero potential and the cycle continues by sweeping between positive (+1.5 V) to negative (−0.5 V) voltage with a scan rate of 0.1 V/s.

### Antibacterial Properties

PDA-coated meshes and control PP mesh without PDA coating were placed in 12-well tissue culture plates and sterilized using ethylene oxide. The antibacterial activities of the PDA coatings were evaluated following published protocol with some modifications (Häntzschel et al., 2009). Both *S. epi* and *E. coli* grown on agar plates were diluted by sterile PBS to the desired concentration [approximately 1–3 × 10^5^ colony forming unit (CFU)/mL]. Two milliliters of this bacteria solution was added to each well, and the plates were incubated at 37°C for 24 h. At a given time point (3, 6, and 24 h), a 10-μl loop was immersed into the mixture and streaked onto agar plates, which were further incubated for 24–48 h. The agar plates with colonies were photographed, and the bacteria colonies were counted manually. The Log reduction values (LRV) were calculated following the equation below (Clement et al., 2002):

(1) $$\begin{array}{l} \text{LRV}={\text{Log}}_{10}\left(\frac{\text{A}}{\text{B}}\right) \end{array}$$

where A is the colony number formed from the bacteria exposed to the uncoated mesh (control) and B is the number of colonies formed from the bacteria exposed to the PDA-coated mesh. The experiment has been performed in triplicate, and the results are reported as mean ± SD.

### Live/Dead Cell Viability Assay

PDA-coated meshes and PP mesh without PDA coating were sterilized using ethylene oxide. The meshes were placed vertically along the wall of the well so that the cells can be in direct contact with the H_2_O_2_ released from the PDA coatings. Biocompatibility of the PDA-coated meshes was investigated using live/dead cell viability assay according to previously published protocol (Meng et al., 2017). L929 rat dermal fibroblasts were seeded into a 12-well plate with a density of 1 × 10^4^ cell/well and incubated at 37°C in a 5% CO_2_ humidified incubator for 24 h to obtain a monolayer of cells. After replacing the culture medium by 2 ml of fresh medium, the meshes were introduced into the 12-well plate and incubated for an additional 24 h. Cells were then stained with calcine and ethidium bromide with a ratio of 1:1,000 in PBS for 15 min at room temperature. An EVOS microscope and software (Thermo Fisher Scientific, Rockford, IL) was used to image and overlay the fluorescence images. Cells were counted using ImageJ to determine the number of live (green) and dead (red) cells. The relative cell viability was calculated with the following formulas:

(2) $$\begin{array}{l} \text{Relative Cell Viability}=\frac{\text{Number of live cells}}{\text{Number of live cells }+\text{ Number of dead cells}}\\ \;\times 100\% \end{array}$$

Images were taken at the middle of the well and near the edge of the culture well (directly beneath the mesh) to compare the localized differences in the cell viability. Additional experiment was carried out in the presence of 20–50 U/ml of bovine liver catalase in the cell culture media to determine if H_2_O_2_ is the source of cytotoxicity. Experiments were performed in triplicate.

### Statistical Analysis

Statistical analyses were performed using SigmaPlot. One-way analysis of variance (ANOVA) with Holm-Sidak method and Student *t*-test were used for comparing means of multiple groups and two groups, respectively, using a *p*-value of 0.05.

## Results

### Determination of H_2_O_2_ Concentration

**Figure 2:**
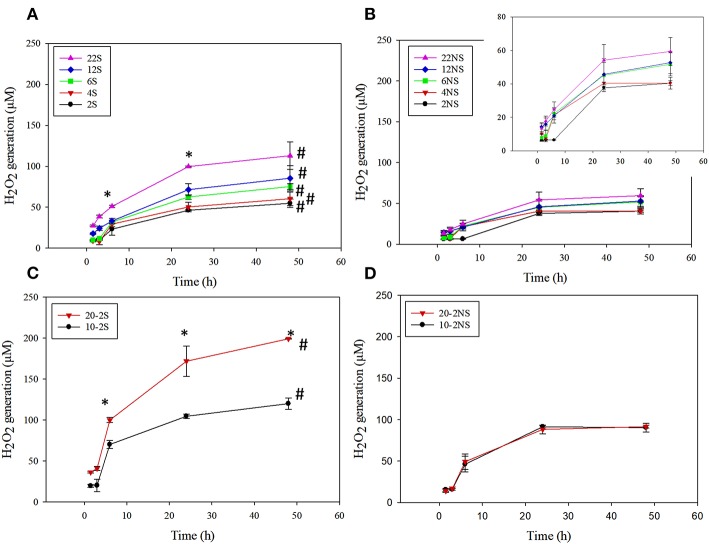
H_2_O_2_ generation from PDA-coated mesh incubated in PBS (pH = 7.4) at 37°C. Coated meshes were prepared in one step with shaking **(A)**, one step without shaking **(B)**, two steps with shaking **(C)**, and two steps without shaking **(D)**. Data are shown as mean ± standard deviation of three independent samples. *p < 0.05 when compared to other PDA coated samples. #*p* < 0.05 when compare to the not-shaken sample prepared with the same dopamine concentration.

PDA-coated meshes started to generate H_2_O_2_ once they were submerged in PBS. The effect of dopamine concentration added and the method of preparation (i.e., shaking vs. non-shaking and one-step vs. two-step) on H_2_O_2_ generation were evaluated (Figure 2). For one-step shaken samples, the concentration of H_2_O_2_ generation increased with dopamine concentration, with 22S exhibiting the highest amount of H_2_O_2_ (112.99 μM) generation after 48 h (Figure 2A). On the other hand, one-step non-shaken samples did not demonstrate the same concentration dependence and the amount of H_2_O_2_ generated by all samples averaged around 50 μM regardless of dopamine concentration (Figure 2B). Most interestingly, the two-step shaken coatings (i.e., 20-2S and 10-2S) exhibited significantly higher H_2_O_2_ generation when compared to their corresponding one-step shaken (i.e., 22S and 12S, respectively) and two-step non-shaken coatings (i.e., 20-2NS and 10-2NS, respectively), even though all these samples were prepared with the same total amount of dopamine during the coating process (i.e., combined 22 mg of dopamine). 20-2S generated over 200 μM of H_2_O_2_ after 48 h, which was 1.7–2 times higher when compared to those generated by 22S and 20-2NS. These results clearly indicated that the combination of shaking during coating preparation and a two-step process created coatings with increased ability to be oxidized and generate H_2_O_2_. The placement of the PP mesh during the coating process also contributed to H_2_O_2_ generation. The mesh placed vertically along the wall of the well exhibited higher H_2_O_2_ generation when compared to mesh that was placed on the bottom of the well regardless of shaking (Figure S2).

20-2S generated the highest amount of H_2_O_2_ among all the coating formulations. As such, 20-2S has been selected for further characterization and antibacterial experiment. Results obtained for 20-2S were compared with 22S, 22NS, and 20-2NS samples, given that all these formulations were prepared using the same quantity of dopamine (i.e., 22 mg) during the coating process.

### Chemical Analysis of the PDA-Coated Meshes

**Figure 3:**
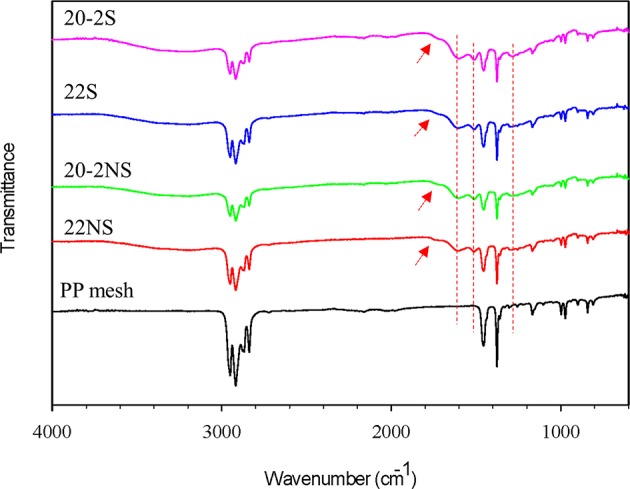
FTIR spectra of PDA-coated PP meshes and uncoated PP mesh. The peaks at 1,303, 1,509, and 1,602 cm^−1^ are attributed to C–N stretching of indole ring, C=N of indole amine, and C=C of the benzene ring (dashed red lines). The peak at 1,723 cm^−1^ is attributed to C=O of quinone (red arrows).

ATR-FTIR spectra confirmed the formation of PDA coatings and the presence of the expected chemical functionalities on the surface of the mesh samples (Figure 3 and Figure S3). All the mesh samples exhibited peaks associated with PP such as 2,916 cm^−1^ (C–H bands), 1,457 cm^−1^ (–CH_2_), and 1,379 cm^−1^ (–CH_3_) (Krylova and Dukštienė, 2013). Spectra of all the PDA-coated meshes contained additional peaks at 1,509 cm^−1^ (C = N of indole amine), 1,602 cm^−1^ (C=C of benzene ring), 1,723 cm^−1^ (C=O of quinone), and 3,100–3,700 cm^−1^ (N–H and O–H stretch vibration) (Patel et al., 2018). There was no apparent differences in the ATR-FTIR spectra for coatings that were prepared differently (i.e., non-shaken vs. shaken, and one-step vs. two-step).

**Figure 4:**
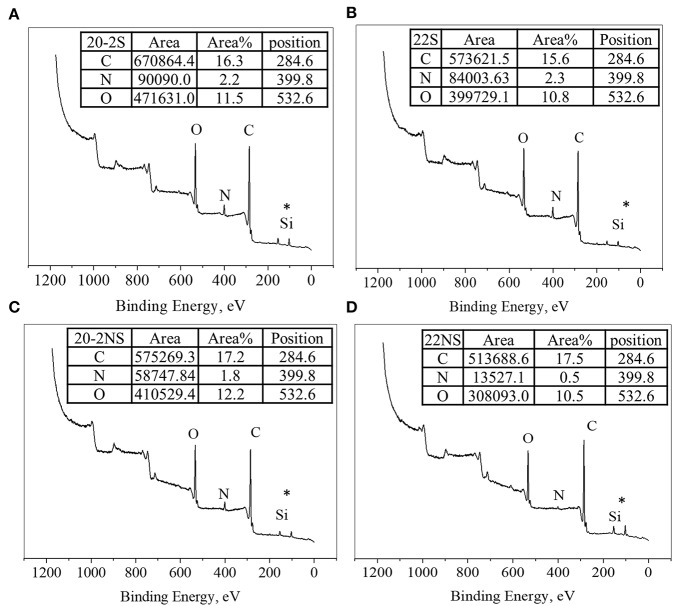
XPS survey scan of PDA-coated PP mesh for 20-2S **(A)**, 22S **(B)**, 20-2NS **(C)**, and 22NS **(D)**. *Si peak is attributed to the SiO_2_ surface of the sample holder.

The surface chemical composition of the PDA-coated mesh was evaluated using XPS (Figure 4). All samples exhibited peaks corresponding to C1s (284.6 eV), N1s (399.8 eV), and O1s (533.6 eV) on their wide scan XPS spectra as expected based on previously published results on PDA coatings (Luo, 2017; Patel et al., 2018). All spectra also contain a Si peak, which corresponded to the silicon dioxide (SiO_2_) at 102.2 eV binding energy on the surface of the sample holder used for the experiment. The elemental percentage obtained from survey scan XPS analysis is shown in Table 1. The shaken samples exhibited higher N content (~6% for 20-2S and 22S) in comparison with the non-shaken samples (5 and 1% for 20-2S and 22S, respectively). The N/C ratios for 20-2S and 22S were 0.08 and 0.09, respectively, which are close to the theoretical N/C value of dopamine (~0.125) (Lee et al., 2007). However, N/C ratios for the non-shaken samples were significantly lower (0.06 and 0.02 for 20-2NS and 22NS, respectively), potentially due to reduced content of PDA.

**Table 1:** XPS elemental percentage of PDA-coated PP mesh.

**Samples**	**C**	**N**	**O**	**Si**	**N/C**
20-2S	69.10	5.76	20.22	4.92	0.08
22S	67.83	6.16	19.68	6.33	0.09
20-2NS	70.50	4.47	20.94	4.09	0.06
22NS	74.33	1.21	18.56	5.90	0.02

High-resolution XPS spectra of the C1s region were deconvoluted for all the PDA-coated mesh samples (Figure S4) and showed three peaks indicating three types of carbon including C–C (284.6 eV), C–O/C–N (286.0–286.3 eV), and C=O (287.6–287.8 eV) (Bernsmann et al., 2009; Luo, 2017; Patel et al., 2018). The C–C peak at 284.6 eV is attributed to aliphatic carbons. Based on the area percentage listed, the shaken samples exhibited a higher percentage of C=O in comparison to their corresponding non-shaken samples. This is potentially due to extended oxidation of the underlying PDA layer as a result of agitation, which facilitates the supply of O_2_ during PDA coating (Bernsmann et al., 2010). Similarly, the high-resolution XPS spectra of the N1s region were deconvoluted into three peaks (Figure S5), which were attributed to aromatic tertiary nitrogen (= N–R, 398.0–398.7 eV), aromatic secondary amine (R_2_-NH, 399.6–400 eV), and primary amine (RNH_2_, 401.4–401.9 eV) (Bernsmann et al., 2009; Patel et al., 2018). The area percentage indicated that the main contribution of N1s spectra for all samples comes from R_2_-NH, indicating that the cyclization of dopamine occurred during the coating process and less open-chain dopamine remained on the surface (Ding et al., 2014).

### Coating Morphology and Topography

**Figure 5:**
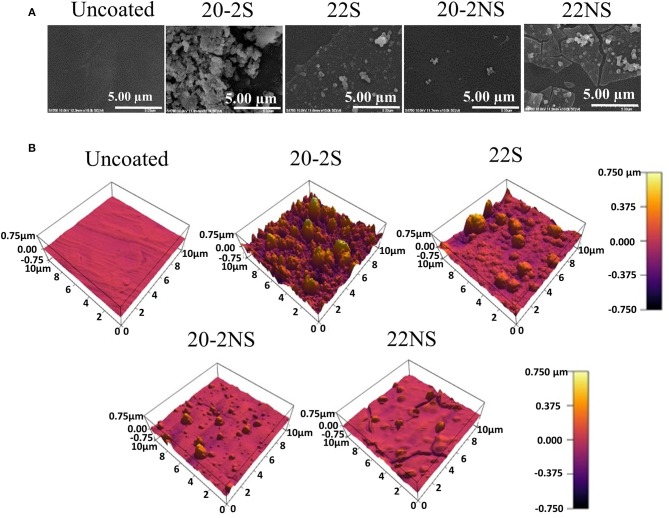
FE-SEM **(A)** and AFM images **(B)** of uncoated mesh and PDA-coated meshes.

FE-SEM was used to visualize the morphology of PP mesh with and without PDA coating (Figure 5A). The uncoated mesh revealed a smooth surface as expected. PDA-coated mesh contained nano-scaled particles and their aggregates on the surface of the mesh fiber. The shaken samples exhibited higher concentration of these nanoparticle aggregates when comparing to the non-shaken samples. During the process of dopamine polymerization, PDA nanoparticles formed in the solution, which may have deposited onto the coating surface during the coating process (Su et al., 2016). Particularly, 20-2S revealed the highest densities of these nanoparticle aggregates when compared to other coatings. 22NS exhibited an unstable coating with cracks throughout the whole sample. This is potentially due to the compliance mismatch between the rigid PDA (elastic modulus of 10 GPa) coated on a softer polymer PP (modulus of 1.3 GPa) (Yang et al., 2012).

**Figure 6:**
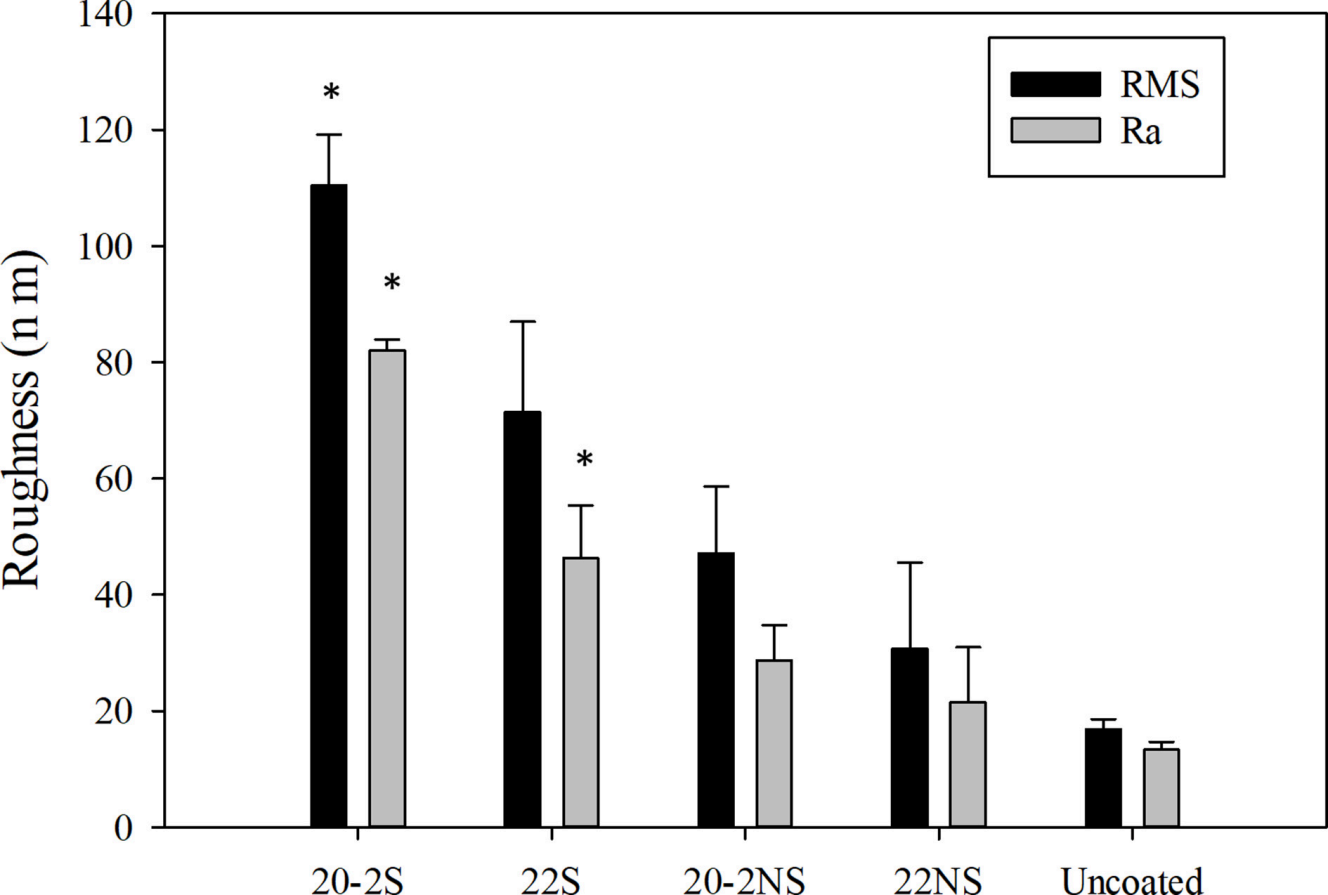
Surface roughness of PDA-coated meshes and uncoated PP mesh determined using AFM. Root mean squared roughness (RMS) and average roughness (Ra) values were reported for the 10 × 10 μm^2^ images. Data are shown as mean ± standard deviation of three independent measurements. *p < 0.05 when compared to other samples including uncoated and PDA-coated.

The surface topography determined using AFM (Figure 5B) was in agreement with the FE-SEM images. All the PDA-coated meshes revealed micron- and nano-scaled surface features that were not present on the smooth, uncoated PP mesh. The shaken samples displayed more of these surface features when compared to their non-shaken counterparts. 20-2S displayed the highest concentration of these surface features amongst all the samples tested, which corresponded to a surface with the highest measured RMS and Ra values (110 nm and 82 nm, respectively) (Figure 6). Although roughness values have not been previously reported for shaken samples (Su et al., 2016), the reported RMS values obtained from the PDA-coated titanium oxide (TiO_2_) surface (non-shaken sample) was 2.5–20 nm (Ding et al., 2014), which is close to our data for 22NS (RMS 30 nm). The AFM scan of 22NS also exhibits surface cracks that were observed using FE-SEM. Both FE-SEM and AFM results indicated the two-step coating process and gentle shaking combined to create a PDA coating with an elevated density of nano-scaled surface roughness.

### 3D Profilometer

The thickness of the PDA coating deposited through the shaking-assisted method was significantly higher than that of the non-shaken samples (Figure S6). The thickness measured for 20-2S averaged around 72 nm, which is significantly higher than other PDA-coated samples. For 22S, the coating thickness averaged around 34 nm, which was roughly half of what was measured for 20-2S. The thickness for 22S was comparable with the thickness (50 nm) reported for coating formed from agitation of the dopamine solution (Lee et al., 2007). This indicates that having a base layer of PDA promoted more PDA deposition in the second coating step. Additionally, successive immersion of the substrate in freshly prepared dopamine solution (multi-step coating) significantly increased the film thickness (Bernsmann et al., 2011) depending on the number of steps and the duration of immersion (Bernsmann et al., 2010). The non-shaken samples (20-2NS and 22NS) revealed low coating thickness of around 9 nm, which is comparable to PDA-coated TiO_2_ substrates (15 nm) formed under static conditions (Ding et al., 2014). This could be due to the limited O_2_ supply within the dopamine solution, which reduced dopamine oxidation and film formation. These data indicate that the combination of two-step coating method and gentle shaking resulted in more deposition of PDA onto the substrate surface.

### Contact Angle

The water contact angle of the uncoated PP sheet was 98.3°, which decreased to 62° with one-step PDA coating (22NS) (Figure S7). This value is consistent with previously reported values of 50–70° for PDA coatings (Ding et al., 2014; Su et al., 2016). Applying the second step of PDA coating (i.e., 20-2NS) further reduced the contact angle to 42°, corresponding to more PDA deposition. Gentle shaking resulted in further increase in surface hydrophilicity and 20-2S exhibited the lowest contact angle (32.3°) among all the samples tested. Shaking-assisted coating has been previously reported to increase the hydrophilicity of PDA coating (Su et al., 2016). The combination of increased PDA deposition and nano-scaled surface roughness likely contributed to this increased wettability (Su et al., 2016).

### Cyclic Voltammetry

**Figure 7:**
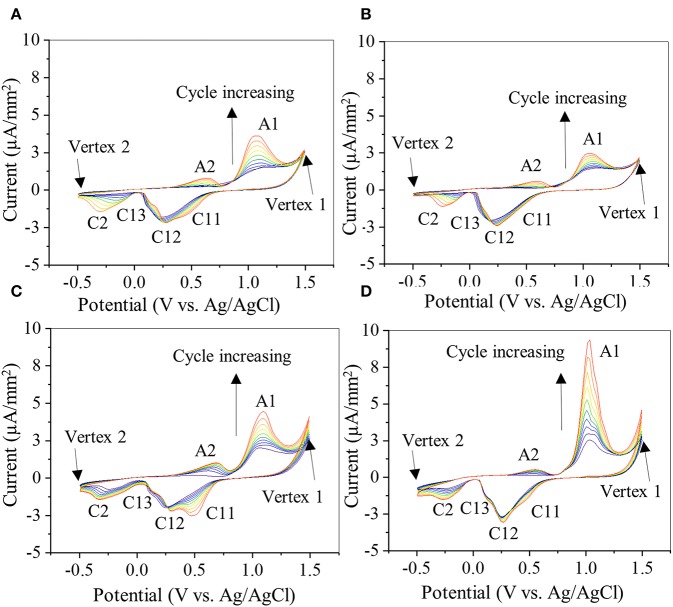
Cyclic voltammogram of gold electrode coated with 20-2S **(A)**, 22S **(B)**, 20-2NS **(C)**, and 22NS **(D)** scanned at rate of 0.1 V/s for 10 cycles. Anodic peaks are denoted by A1 and A2 and cathode peaks are denoted by C11, C12, C13, and C2. Current level is normalized by the area of the gold electrode coated with PDA.

CV was used to investigate the oxidation–reduction (redox) activity of the PDA-coated gold electrode (Figure 7). The bare gold electrode exhibited an anodic and a cathodic peak at +1.13 and +0.57 V, respectively (Figure S1B), corresponding to oxidation and reduction of the aqueous buffer solution (Rahaman et al., 2014). The same potential window was used to investigate the electrochemical activity of PDA coating on gold electrode for up to 10 CV cycles (Figure 7). All PDA coatings exhibited similar anodic and cathodic peaks (Figures 7A–D). Typically, during the first cycle, starting from zero potential, an anodic peak (A1) appeared at +1.06 V in the forward scan, which is attributed to the oxidation of catechol to its quinone form (Amiri et al., 2016; Motin et al., 2017; Salazar et al., 2019). In the reverse scan, cathodic peaks C12 (+0.25 V) and C2 (−0.22 V) were observed, which are attributed to the reduction of dopamine quinone and dopamine chrome, respectively (Nematollahi et al., 2009; Salazar et al., 2019). A new anodic peak (A2) appeared at the beginning of the second cycle centered at +0.57 V, which is attributed to the oxidation of leucodopamine chrome. During successive scanning cycles, the peaks C11 and C13 (centered at +0.47 and +0.11 V, respectively) appeared, which could be attributed to the reduction of dopamine oxidation intermediates, cyclization products, or tetramer generated by both chemical and electrochemical reactions driven by redox-active nature of PDA in each cycle (Nematollahi et al., 2009; Salazar et al., 2019).

There were slight shifts in anodic and cathodic peak potentials during multiple cycle scans, regardless of the formulation. However, the redox reactions were unchanged as the formal potential for redox couple (i.e., the average value of anodic and cathodic peak potentials during each cycle) remained unchanged. Additionally, with each cycle, the current levels of the redox peaks increased, potentially due to increased concentration of the reactants, such as oxidized forms of catechol and the formation of diverse redox products (Randviir et al., 2013). The increase in current could also be due to increased ionic and electron transfer as a result of increased wetting of PDA film by the electrolyte with each CV cycle (Wu et al., 2011). The shaken samples exhibited lower anodic (A1) and cathodic (C12) peak currents (Figures 7A,B) when compared to the non-shaken samples (Figures 7C,D) in each corresponding cycle. For example, in the last cycle, A1 and C12 for 20-2S was 3.11 and 2.12 μA/mm^2^, respectively (Figure 7A), which are lower than those of 20-2NS (4.2 and 2.3 μA/mm^2^ for A1 and C11, respectively, Figure 7C). A similar trend was observed for 22S (2.3 and 2.2 μA/mm^2^, for A1 and C11, respectively, Figure 7B) and 22 NS (9.03 and 2.7 μA/mm^2^ for A1 and C11, respectively, Figure 7D) samples. This may be explained by the thick homogeneous PDA film achieved by the shaken method that limits the diffusion of the ions and results in poor electronic transfer. Similarly, for the non-shaken samples (Figures 7C,D), there is a sharp increase in the current level with each subsequent cycle, indicating that PDA coating was being removed from the gold electrode. This result suggests that non-shaken coatings are less stable when compared to shaken samples. Both FE-SEM and AFM images revealed cracks in 22NS (Figure 5), further confirming the poor stability and conformability of this coating.

### Antibacterial Properties

**Figure 8:**
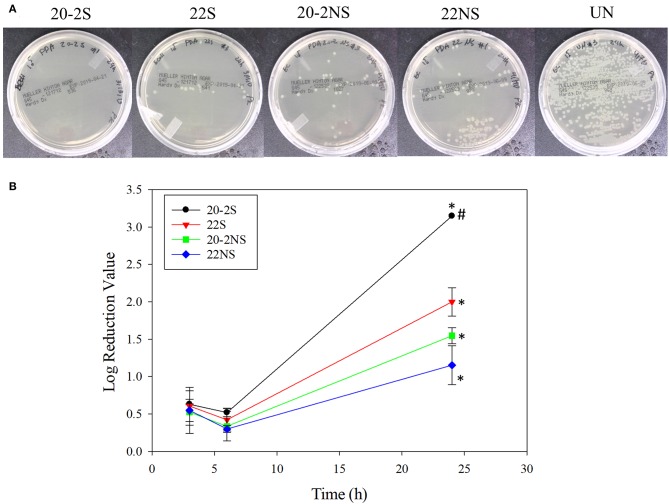
Photograph of the test plates for *E. coli* colonies exposed to PDA-coated meshes and the uncoated mesh control after 24 h **(A)**. Log reduction values for *E. coli* colonies incubated with the PDA-coated meshes, which is normalized by the number of colonies formed by the bacteria exposed to the uncoated mesh **(B)**. Data are shown as mean ± standard deviation of three independent samples. *p < 0.05 when compared to other PDA coated samples. #completely killed bacteria.

**Figure 9:**
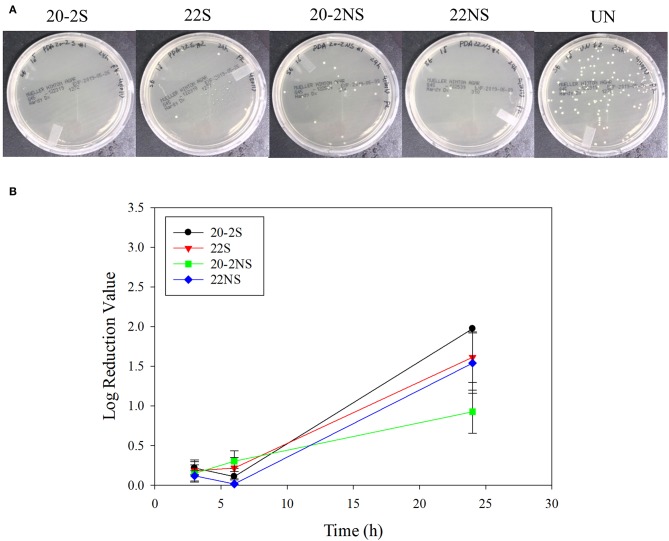
Photograph of the test plates for *S. epi* colonies exposed to PDA-coated meshes and the uncoated mesh control after 24 h **(A)**. Log reduction values for *E. coli* colonies incubated with the PDA-coated meshes, which is normalized by the number of colonies formed by the bacteria exposed to the uncoated mesh **(B)**. Data are shown as mean ± standard deviation of three independent samples.

The antibacterial properties of the PDA-coated meshes were investigated with both gram-positive (*S. epi*) and gram-negative (*E. coli*) bacteria. The photograph of the test plates after 24 h and the LRV for both bacteria are shown in Figures 8, 9. In general, coatings that generated higher concentrations of H_2_O_2_ achieved higher LRV. 20-2S demonstrated the highest LRV and completely killed *E. coli* after 24 h of incubation (100% reduction and LRV ~3.15). 20-2S also achieved 98.9% reduction (LRV ~1.97) when incubated with *S. epi* for 24 h. These gram-positive bacteria are likely more resistant to H_2_O_2_ and required more time or H_2_O_2_ concentration to completely disinfect. 20-2S was a more effective antibacterial coating than both 22S and 20-2NS, indicating that the two-step coating process and gentle shaking both contributed to enhance the antimicrobial property of the coating. In the absence of PDA coating (uncoated PP mesh), both bacteria rapidly replicated over time and reached a very high concentration in 24 h (Figures S8, S9).

### Live/Dead Cell Viability Assay

**Figure 10:**
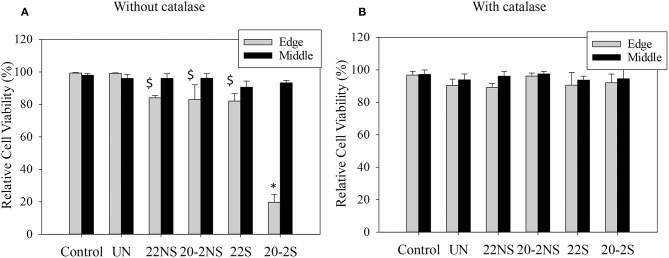
Relative cell viability of L929 fibroblasts directly exposed to PDA-coated mesh, the uncoated mesh (UN), and culture media (control), for cell located at the edge of the culture well (directly beneath the mesh, Edge) and at the middle of the well (Middle) in the absence of **(A)** and in the presence of 20–50 U/ml of catalase **(B)**. Data are shown as mean ± standard deviation of three independent samples. *p < 0.05 when compared to other samples and control. $*p* < 0.05 when compared to control.

Cytotoxicity of PDA-coated meshes and PP mesh without PDA coating was determined by directly exposing the mesh samples to L929 fibroblasts using the live/dead assay. Relative cell viability located at the edge of the culture well and directly beneath the mesh demonstrated lower cell viability when compared to the uncoated PP mesh (Figure 10A, Figure S11). PDA-coated meshes that generated lower amount of H_2_O_2_ (22S, 22NS, and 20-2NS) demonstrated a cell viability of more than 80%. This is in agreement with previous findings that demonstrated PDA coatings to be noncytotoxic toward various types of cells including fibroblast, endothelial, neuron, and osteoblast cells (Ku et al., 2010; Liu et al., 2014). 20-2S generated the highest amount of H_2_O_2_ and exhibited a cell viability of only 20%. The cytotoxicity effect was highly localized near the mesh and cells imaged near the center of the well exhibited viability >90%. Catalase is an enzyme that decomposes H_2_O_2_ into water and O_2_ (Chelikani et al., 2004). In the presence of catalase, no H_2_O_2_ was detected from the PDA-coated meshes (Figure S10) and cell viability was found to be 90% for all samples for cells examined at both locations (Figure 10B, Figure S12). This result indicates that the source of cytotoxicity associated with 20-2S is attributed to the elevated concentration of H_2_O_2_ released from the coating (170–200 μM; Figure 2). Addition of catalase counteracted the cytotoxic effect of H_2_O_2_ and significantly increased cell viability. Additionally, the cytotoxicity effect of H_2_O_2_ released from the PDA-coated mesh was highly localized, potentially due to the instability of H_2_O_2_ (Reznikov et al., 2000) and its short diffusion radii in the culture medium (Takano et al., 2002). The same localized cytotoxicity response has been observed for catechol-containing hydrogels (Meng et al., 2017).

## Discussion

We described a simple two-step, shaking-assisted coating approach to impart PP mesh with antibacterial properties. This combination created a thick conformal coating (~72 nm, Figure S6) with increased nano-scaled surface roughness (RMS value of ~110.43 nm, Figure 6), which generated the highest amount of H_2_O_2_ (more than 200 μM) among all the prepared coatings. During self-polymerization of dopamine, PDA nanoparticles are generated in the solution (Su et al., 2016; Alfieri et al., 2018). The coating deposition was initiated by the formation of radical compounds that further attached to the surface of the substrate (Bernsmann et al., 2009). When a relatively smaller amount of dopamine is available in the solution, the formation of large insoluble aggregates is encouraged, whereas elevated dopamine concentrations promote the formation of the radical compound and encourage film formation (Alfieri et al., 2018). When we employed the two-step coating process, a relatively large concentration of dopamine (20 mg ml^−1^) was used to create an underlying PDA primer. Shaking the solution during coating potentially promoted catechol oxidation in creating a thicker film (Su et al., 2016; Alfieri et al., 2018). We also observed that dopamine solution appeared darker in color during gentle shaking, suggesting a larger extent of dopamine oxidation. This is potentially due to better gas exchange during shaking to increase molecular oxygen content in the reaction solution, which is necessary for the film formation (Bernsmann et al., 2010, 2011). During the second coating step, a significantly lower concentration of dopamine (2 mg ml^−1^) was utilized, which may have promoted the formation of nanoparticle aggregates. Replacing freshly prepared dopamine solution in the second step allowed for continued PDA film growth by supplying the reaction mixture with unoxidized dopamine (Bernsmann et al., 2009). To increase the deposition of these aggregates onto the mesh surface, gentle shaking was employed to increase the interactions between the aggregates and the underlying PDA primer surface, resulting in a thicker and rougher PDA coating.

Chemical characterization of the PDA-coated mesh confirmed that the chemistry of the film formed through the two-step method is similar to the conventional one-step PDA coating method. FTIR spectra indicated that all four coatings contained similar functionalities associated with PDA including C=N (indole amine), C=C (benzene ring), and C=O (quinone, Figure 3) (Patel et al., 2018). XPS further verified the formation of PDA film with a thickness of at least 9 nm (Figure 4), which is consistent with the results obtained by 3D Profilometer (Figure S6). However, gentle shaking of the coatings during preparation process resulted in higher N/C ratio than the non-shaken coatings, indicating a higher content of PDA and film formation (Figure 4). Additionally, CV analysis also confirmed that the two-step shaking-assisted method exhibited thicker and more stable film when compared to the other coatings, as demonstrated by reduced electron conductivity and stability of the coating (Figure 7). These observed differences are purely associated with the differences in the preparation methods, and directly contributed to different H_2_O_2_ release profile. The thickest and most stable coating prepared through the two-step shaking-assisted approach released the highest concentration of H_2_O_2_ among the coatings tested (Figure 2).

PDA-coated PP mesh continuously generated antibacterial levels of H_2_O_2_ for over 48 h. The catechol moieties that exist in the PDA film autoxidize in the presence of O_2_ at a mildly basic pH and generate ROS such as ${\text{O}}_{2}^{\;\cdot -}$ and H_2_O_2_ as by-products (Figure S13) (Mochizuki et al., 2002; Meng et al., 2015). During this reaction, O_2_ oxidizes catechol to semiquinone and then quinone, and is transformed to a highly reactive ${\text{O}}_{2}^{\cdot -}$ (Mochizuki et al., 2002; Meng et al., 2019). ${\text{O}}_{2}^{\cdot -}$ readily oxidizes catechol and generates H_2_O_2_. Possible reaction between ${\text{O}}_{2}^{\cdot -}$ and proton ions can also result in H_2_O_2_ generation (Sawyer and Valentine, 1981). H_2_O_2_ is an attractive disinfectant because it decomposes into biocompatible degradation products such as water and O_2_ (McDonnell, 2014). When applying a single dose of H_2_O_2_, a significantly higher amount of H_2_O_2_ is required for bacteriostatic (10^3^ μM) (Thomas et al., 1994) and bactericidal (10^6^ μM) (McDonnell, 2007, 2014) effects against both gram-positive (*Streptococcus, Enterococcus*) and gram-negative (*Pseudomonas, Klebsiella, Escherichia*) bacteria. Although PDA-coated mesh only generated around 10^2^ μM of H_2_O_2_, in our previous study, we showed that the sustained release of low doses of H_2_O_2_ likely maintained sufficient antibacterial levels of H_2_O_2_ (Meng et al., 2019). Additionally, ${\text{O}}_{2}^{\cdot -}$ generation may also have contributed to the enhanced antibacterial property. However, the PDA-coated mesh generated an order of magnitude lower H_2_O_2_ when compared to microgels that were modified with dopamine that have not been previously oxidized (Meng et al., 2019). Although our CV experiment confirmed that PDA coatings remained redox active, extensive autoxidation and cross-linking during the coating process decreased PDA's ability to be further oxidized to generate elevated levels of H_2_O_2_.

H_2_O_2_ generated from PDA coating was found to illicit localized cytotoxic toward fibroblasts. However, a simple cell culture medium lacks antioxidant enzymes that may be present naturally in the human body, such as catalase, glutathione peroxidase, and peroxiredoxin, that can detoxify H_2_O_2_ (Schäfer and Werner, 2008; Bryan et al., 2012). Additionally, it has been demonstrated that relatively low concentrations of H_2_O_2_ induced the expression of vascular endothelial growth factor (Sen et al., 2002) and promoted the recruitment and differentiation of M2 macrophage (Meng et al., 2017), which can encourage angiogenesis and tissue regeneration. Many studies have shown PDA coatings to be noncytotoxic toward many types of cells including fibroblast, endothelial, and neuron cells (Ku et al., 2010) and even enhanced adhesion and proliferation of endothelial cells (Luo et al., 2013; Liu et al., 2014). As such, the biocompatibility of the PDA-coated mesh needs to be further determined using a clinically relevant animal model.

Nevertheless, the coating process described here successfully coated inert PP mesh without using expensive laboratory equipment such as a plasma treatment system or organic solvents that may compromise the integrity of the PP mesh. The PDA-coated mesh itself does not contain H_2_O_2_, which makes it easier and less hazardous to store and transport given the reactive and unstable nature of the peroxide (McDonnell, 2014). The PDA-coated mesh was activated to release H_2_O_2_ only when it was hydrated in an aqueous solution. The simplicity in activation is attractive, as surgeons will not need to alter their surgical procedures in order to deploy this biomaterial.

## Conclusion

We successfully modified the conventional PDA coating method by a simple two-step, shaking-assisted coating approach to create an antimicrobial PP mesh tailored for H_2_O_2_ generation. H_2_O_2_ (60–200 μM) was generated from PDA-coated meshes for over 48 h when the mesh was hydrated in PBS. A relatively large concentration of dopamine (20 mg ml^−1^) was used to create a PDA primer layer on the surface of the mesh, which was followed by a second coating step that utilized a significantly lower concentration of dopamine (2 mg ml^−1^) that promoted the formation of a thick PDA film with nano-scaled surface roughness. Gentle shaking the solution during coating process facilitated gas exchange with the atmosphere, which promoted catechol oxidation and the formation of thicker PDA films. The combination of multi-step coating and gentle shaking created PDA films that generated significantly higher H_2_O_2_. H_2_O_2_ generated from the PDA-coated mesh was antibacterial against both gram-positive (*S. epi*) and gram-negative (*E. coli*) bacteria. H_2_O_2_ released from the PDA coating exhibited localized cytotoxicity, which was significantly reduced in the presence of catalase. The simplicity in the coating process and activation for H_2_O_2_ generation makes the PDA-coated mesh a potentially useful biomaterial for preventing microbial infection.

## Data Availability Statement

All datasets generated for this study are included in the manuscript/Supplementary Files.

## Author Contributions

BL and PF conceived the idea, designed the experiments, and wrote the manuscript. PF also performed experiments. EP and KT prepared samples and performed FOX assay. MB performed experiments and wrote texts related to CV experiment and helped to analyze XPS data. RP performed FE-SEM, ATR-FTIR, and biocompatibility experiments. MT and KP performed and wrote the section associated with AFM experiment and helped with XPS analysis. YG performed contact angle measurements and was supervised by LP. CK prepared gold electrodes and performed thickness measurement. BL supervised the whole project. All authors contributed to analyzing the data and writing the manuscript.

### Conflict of Interest

The authors declare that the research was conducted in the absence of any commercial or financial relationships that could be construed as a potential conflict of interest.

## Abbreviations

20-2S: PDA-coated sample prepared via two-step coating process with gentle shaking using 20 and 2 mg ml^−1^ dopamine HCl
22S: PDA-coated sample prepared via one-step coating process with gentle shaking using 22 mg ml^−1^ dopamine HCl
20-2NS: PDA-coated sample prepared via two-step coating process under non-shaking (static) condition using 20 and 2 mg ml^−1^ dopamine HCl
22NS: PDA-coated sample prepared via one-step coating process under non-shaking (static) solution condition using 22 mg ml^−1^ dopamine HCl.
