# Supplementary material for: Antibacterial Properties of Mussel-Inspired Polydopamine Coatings Prepared by a Simple Two-Step Shaking-Assisted Method
Source: Front Chem. 2019 Sep 25;7:631. doi: 10.3389/fchem.2019.00631 (PMC6773806; doi:10.3389/fchem.2019.00631)
Supplement: Supplementary file 1 [file Data_Sheet_1.PDF]

Supplementary information

Antibacterial Properties of Mussel-Inspired Polydopamine Coatings Prepared by Simple Two-Step Shaking-Assisted Method

Pegah Kord Forooshani[1], Elizabeth Polega[1], Kevin Thomson[1], Md. Saleh Akram Bhuiyan[1], Rattapol Pinnaratip[1], Mikhail Trought[2], Chito Kendrick[3], Yuesheng Gao[4], Kathryn A. Perrine[2], Lei Pan[4], Bruce P. Lee[1*]

[1]Department of Biomedical Engineering, Michigan Technological University, Houghton, MI, USA

[2]Department of Chemistry, Michigan Technological University, Houghton, MI, USA

[3]Department of Electrical Engineering, Michigan Technological University, Houghton, MI, USA

[4]Department of Chemical Engineering, Michigan Technological University, Houghton, MI, USA

*** Correspondence:**
Corresponding Author
bplee@mtu.edu

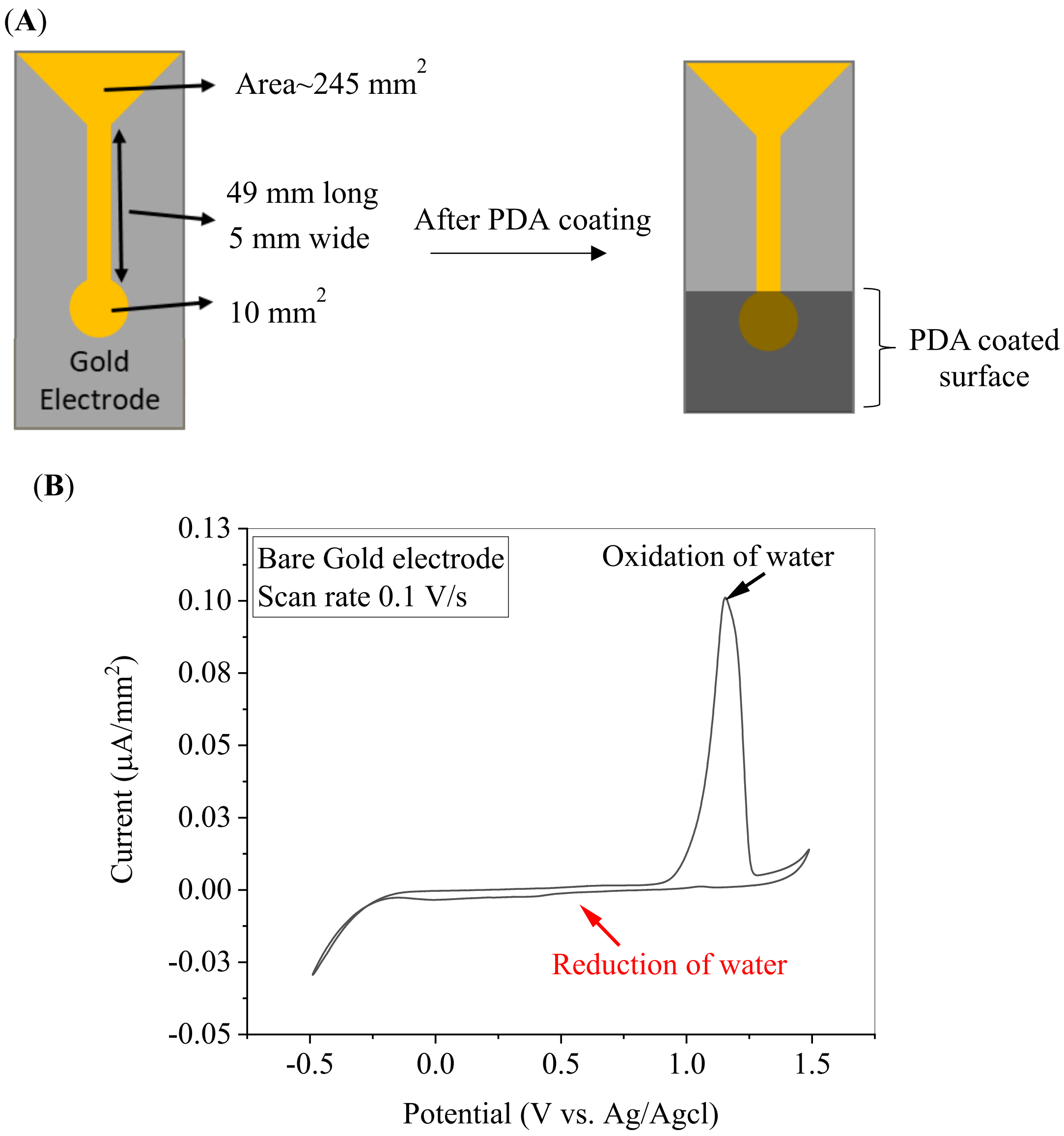

Figure S1. Schematic representation of the sputtered gold electrode before and after PDA coating (A) and cyclic voltammogram of bare sputtered gold electrode (B).

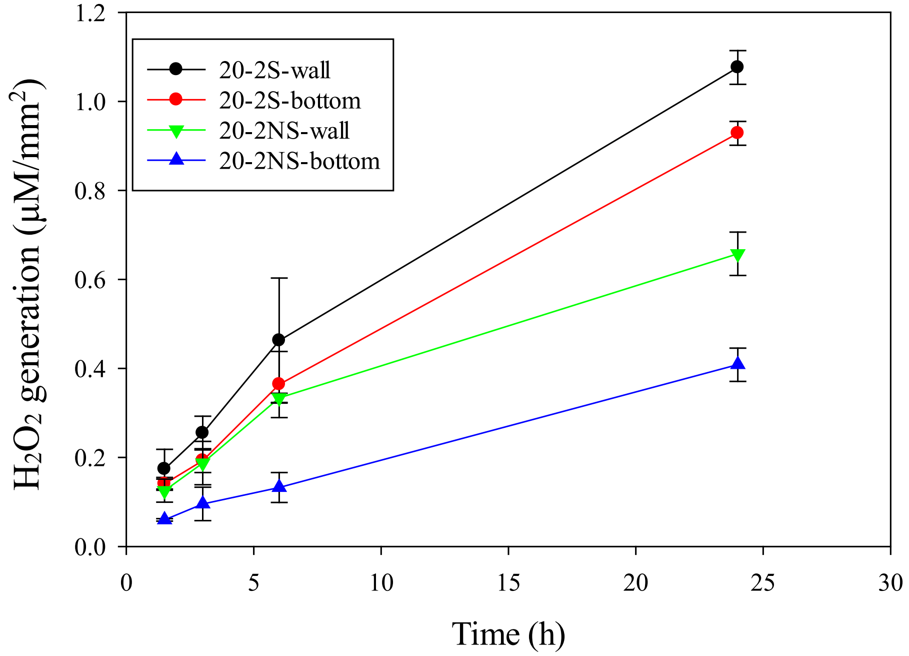

Figure S2. H_2O_2 generation from the PDA-coated meshes that were either affixed at the bottom or around the circumference of wall of a well in the 12-well cell culture plate. H_2O_2 generation was normalized to the surface area of the PDA-coated mesh incubated in PBS (pH = 7.4) at 37°C.

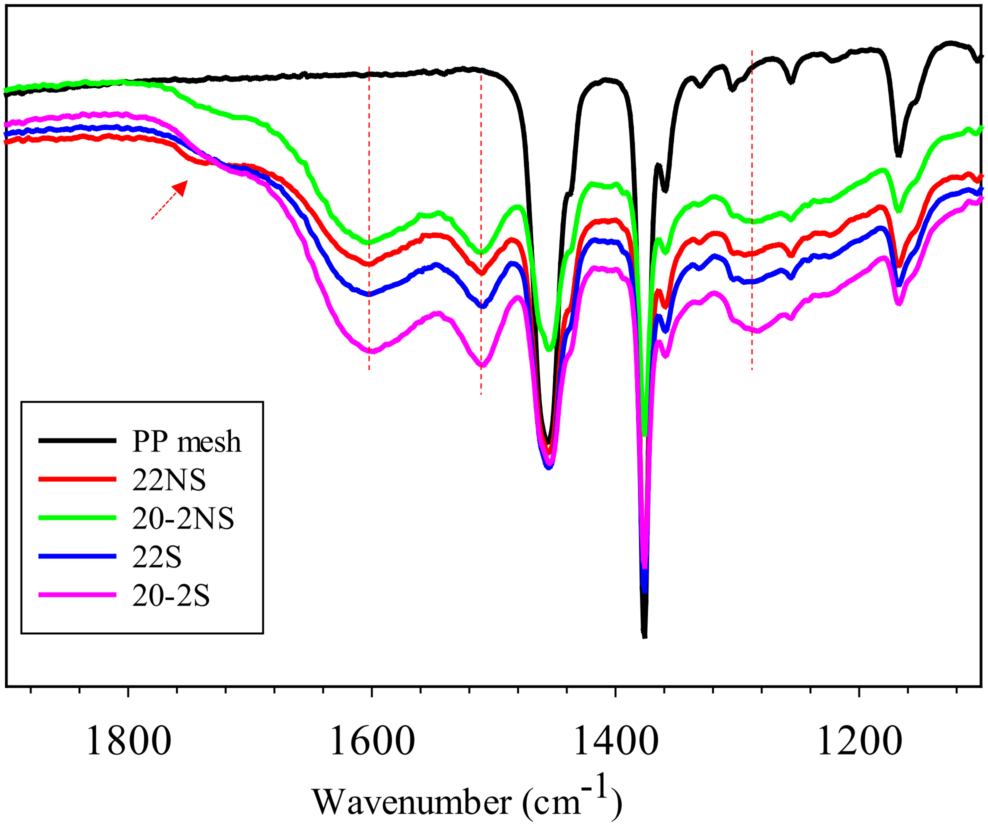

Figure S3. FTIR spectra of PDA-coated PP meshes and uncoated PP mesh focusing on the region of 2000-1100 cm^{-1}. The peaks at 1303, 1509 and 1602 cm^{-1} are attributed to C-N stretching of indole ring, C=N of indole amine and C=C of the benzene ring, respectively (dashed red lines). The peak at 1723 cm^{-1} is attributed to C=O of quinone (red arrow).

20-2S	FWHM	Area	Area %	Position
C-C	1.8	23230.8	78.5	284.6
C-O/C-N	1.8	5355.9	18.1	286.1
C=O	1.5	1012.5	3.4	287.8

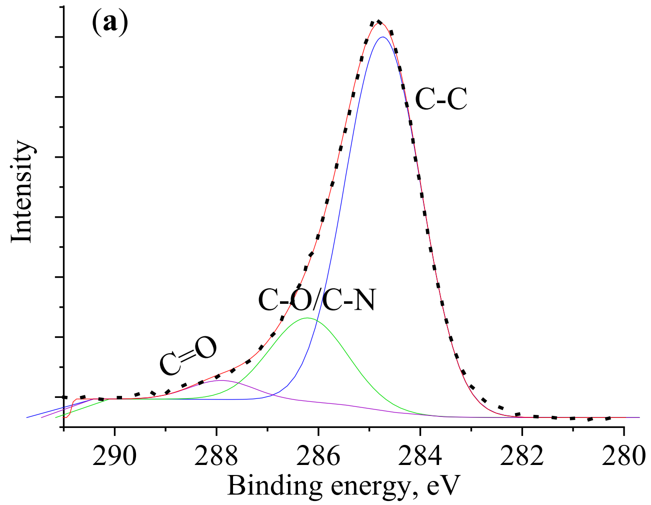

22S	FWHM	Area	Area %	Position
C-C	1.6	16092.3	70.5	284.6
C-O/C-N	1.7	6044.9	26.4	286.0
C=O	1.4	699.4	3.1	287.6

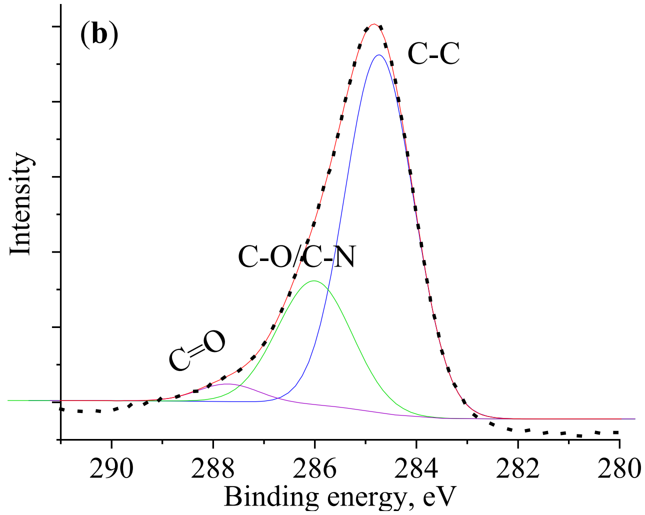

20-2NS	FWHM	Area	Area %	Position
C-C	1.7	16770.6	74.5	284.6
C-O/C-N	1.7	5343.4	23.7	286.0
C=O	1.2	403.1	1.8	287.6

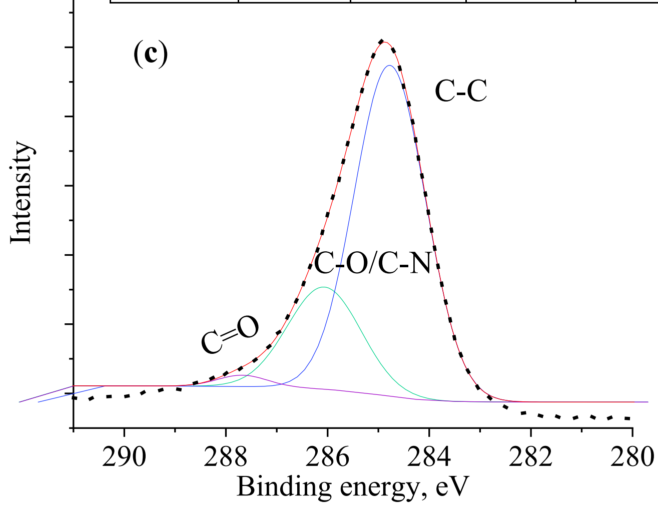

22NS	FWHM	Area	Area %	Position
C-C	1.8	21723.0	90.5	284.6
C-O/C-N	1.3	1672.0	7.0	286.3
C=O	1.3	608.3	2.5	287.7

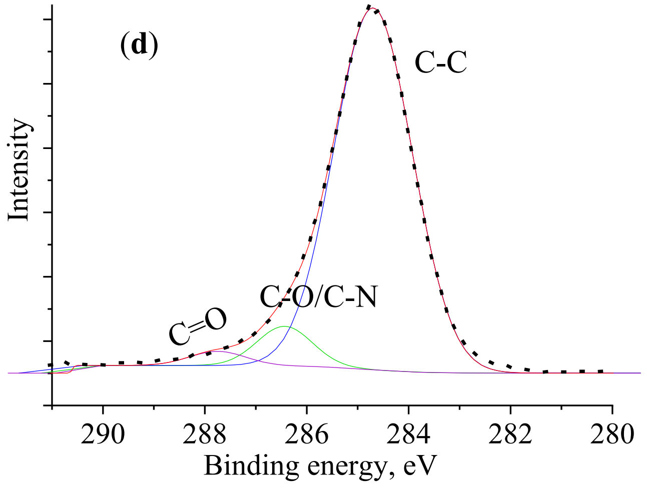

Figure S4. High resolution spectra for C1s peaks (black dotted line) for 20-2S (a), 22S (b), 20-2NS (c), and 22NS (d).

20-2S	FWHM	Area	Area %	Position
=N-R	1.6	238.12	8.6	398.1
R_2NH	1.8	2081.7	74.8	399.9
RNH_2	1.8	462.0	16.6	401.5

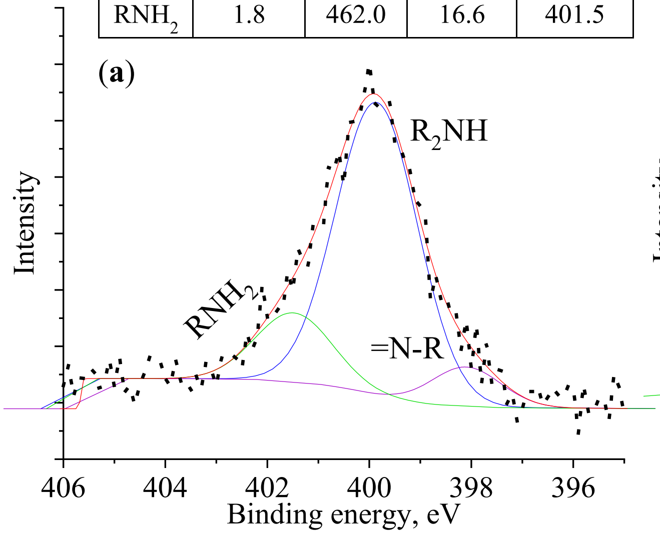

22S	FWHM	Area	Area %	Position
=N-R	1.5	348.3	14.3	398.7
R_2NH	1.6	1515.2	62.3	400.0
RNH_2	1.9	570.3	23.4	401.9

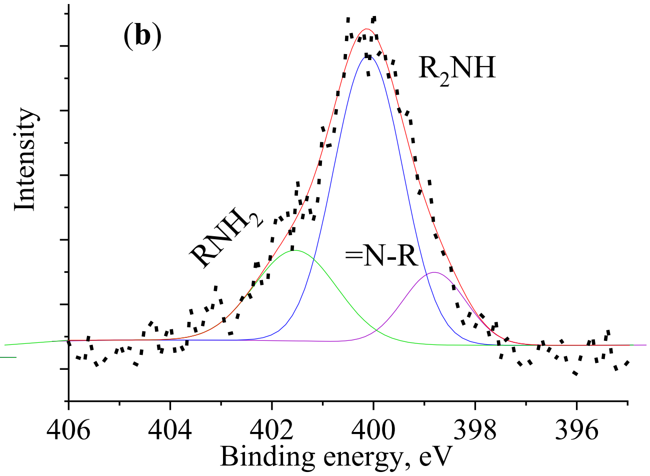

20-2NS	FWHM	Area	Area %	Position
=N-R	1.6	227.1	9.7	398.1
R_2NH	1.8	1630.3	69.7	400.0
RNH_2	2.1	481.3	20.6	401.5

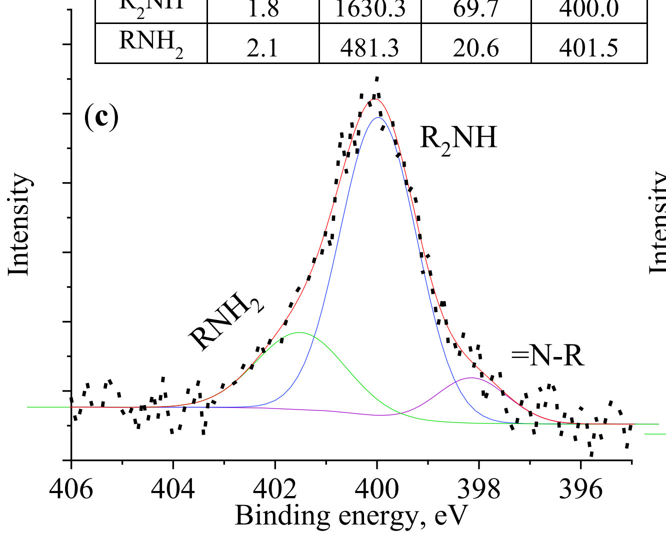

22NS	FWHM	Area	Area %	Position
=N-R	2.1	275.4	12.8	398.0
R_2NH	1.7	486.3	78.2	399.6
RNH_2	2.5	219.7	9.1	401.4

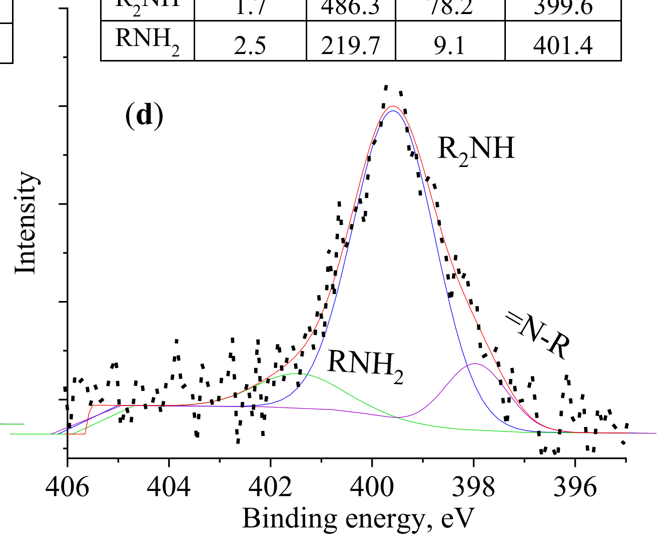

Figure S5. High resolution spectra N1s peaks (black dotted line) for 20-2S (a), 22S (b), 20-2NS (c), and 22NS (d).

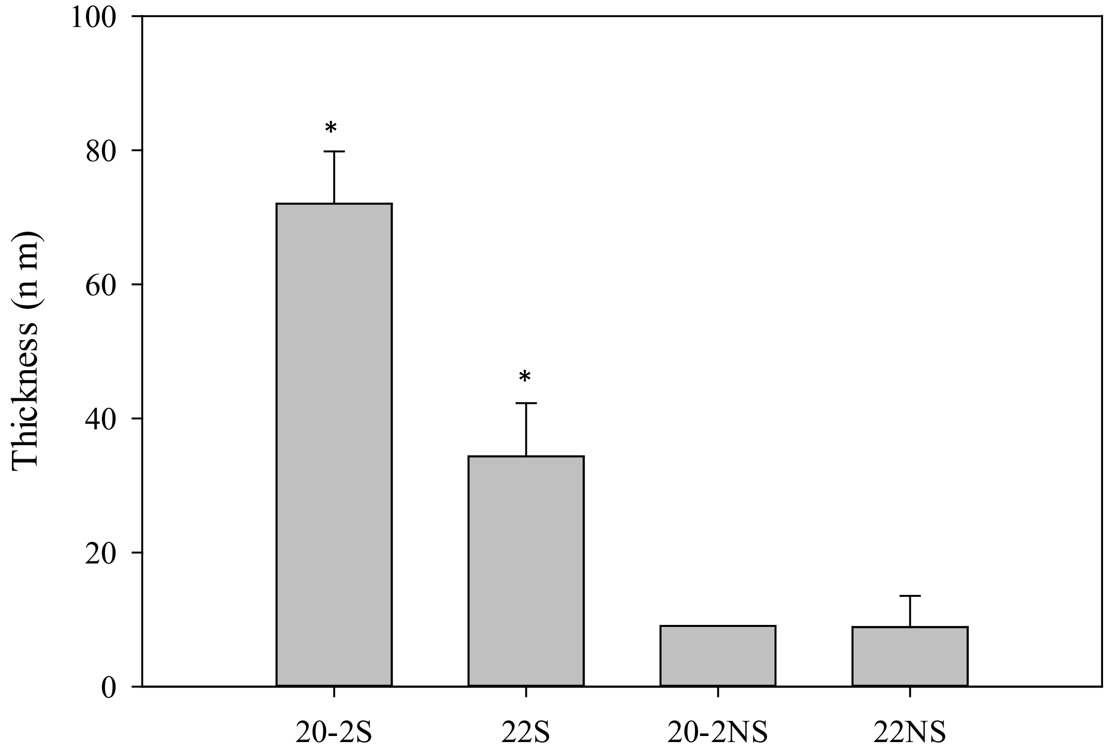

Figure S6. Average thickness of PDA coated on glass slides. Data is shown as mean ± standard deviation of three independent measurements. * $p < 0.05$ when compared to other PDA coated samples.

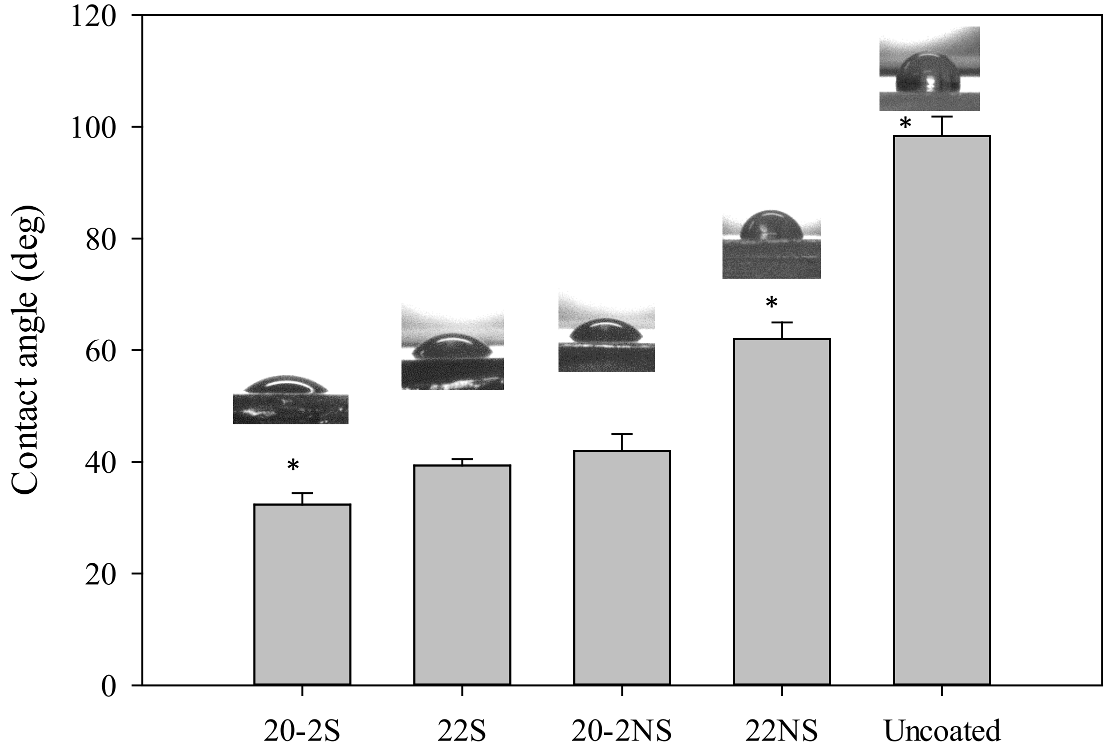

Figure S7. Contact angle measurements and corresponding photograph of the water droplet on the surface of the PDA-coated PP sheets. Data is shown as mean ± standard deviation of three independent measurements. * $p < 0.05$ when compared to other samples.

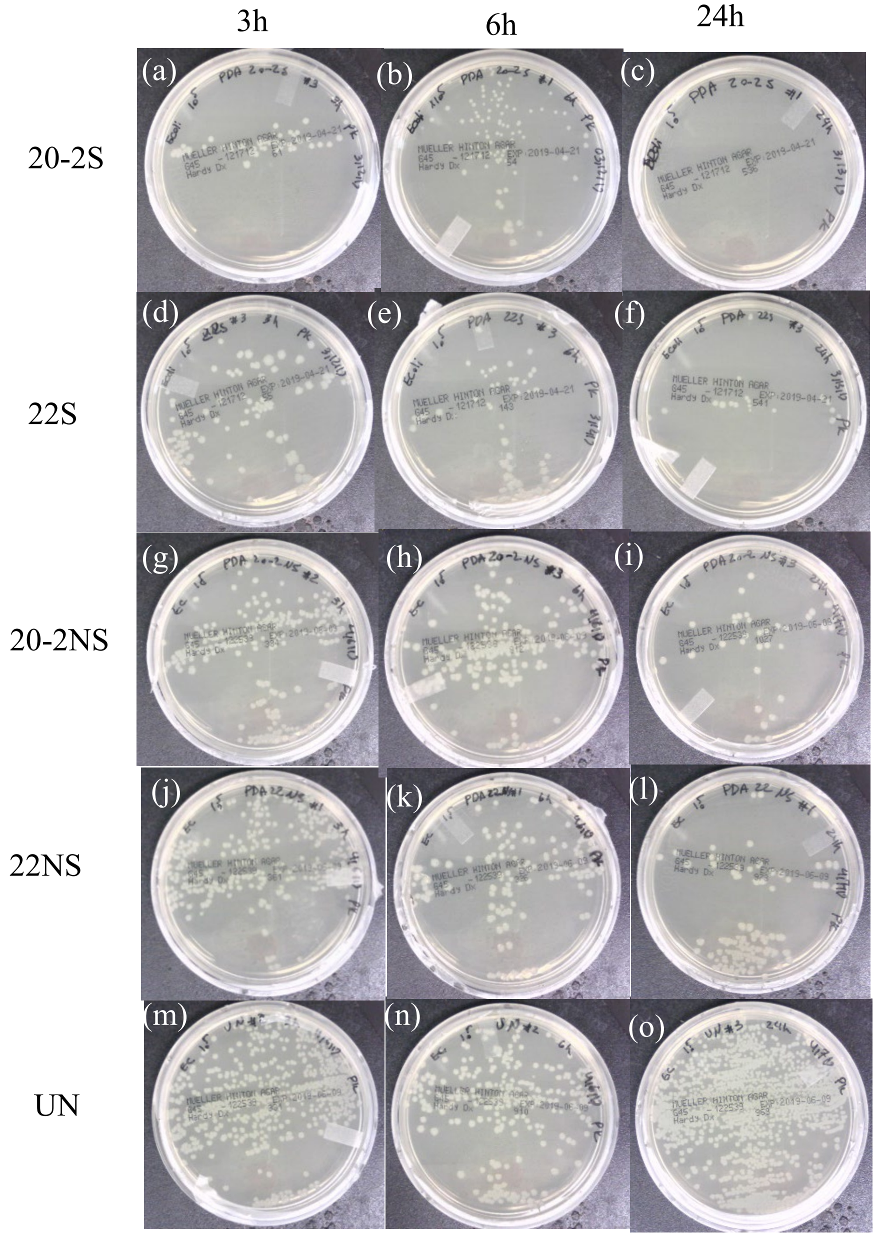

Figure S8. Photograph of the test plates with *E. coli* colonies exposed to 20-2S (a-c), 22S (d-f), 20-2NS (g-i), 22NS (j-l), and the uncoated mesh control (UN, m-o) after 3, 6, and 24h.

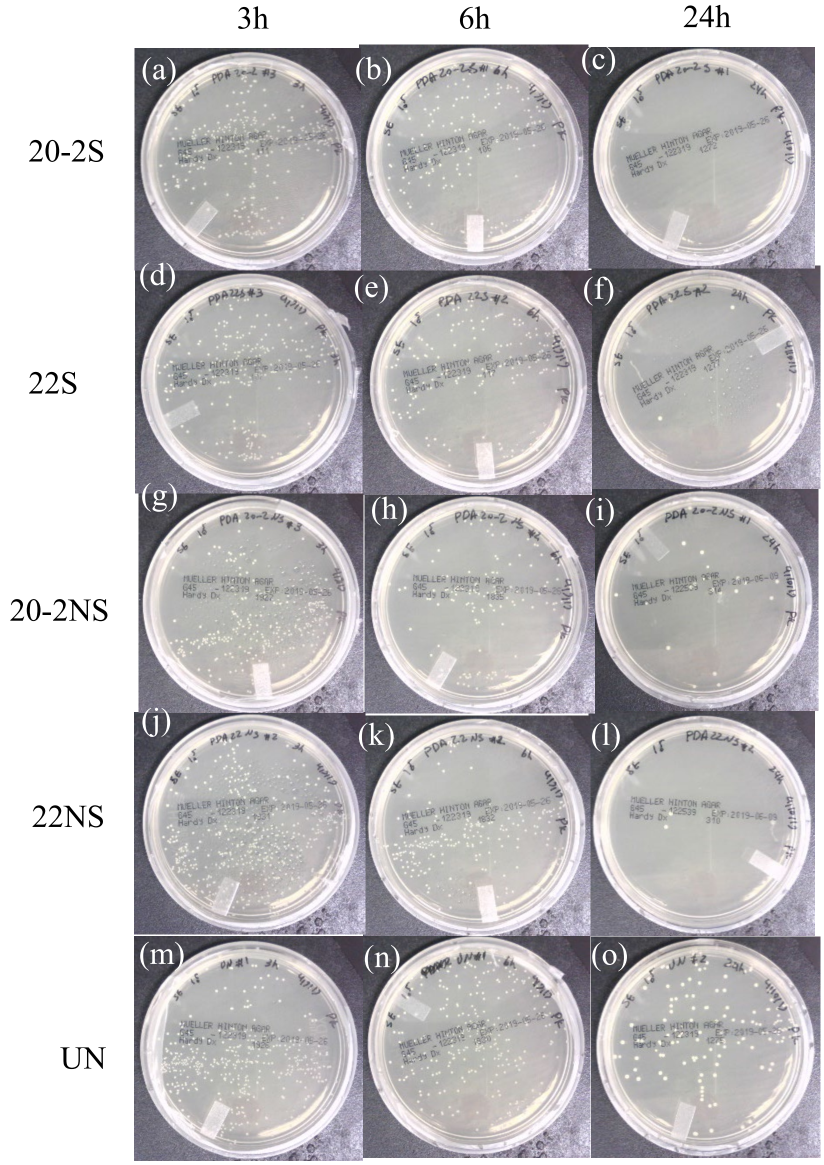

Figure S9. Photograph of the test plates with *S. epi* colonies exposed to 20-2S (a-c), 22S (d-f), 20-2NS (g-i), 22NS (j-l), and the uncoated mesh control (UN, m-o) after 3, 6, and 24h.

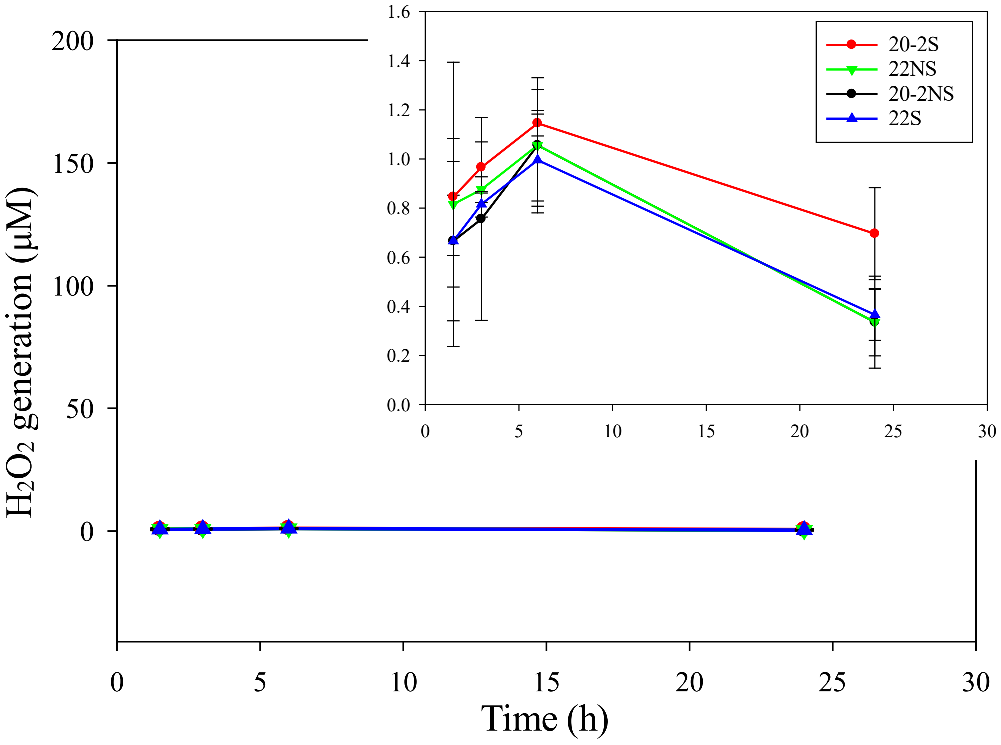

Figure S10. H_2O_2 generation from the PDA-coated meshes in the presence of 40-100 U/ml catalase.

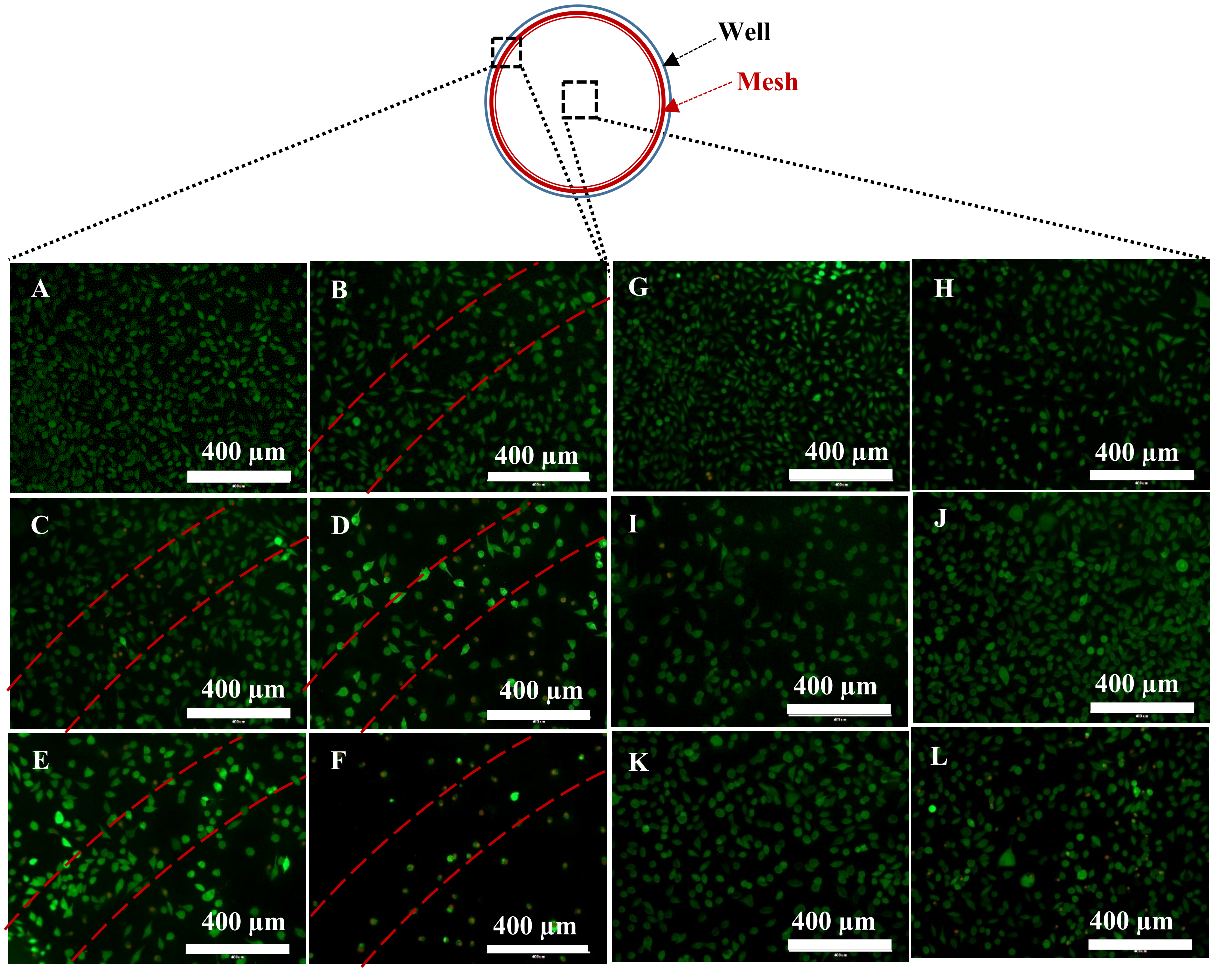

Figure S11. Images of live/dead staining of L929 fibroblast located at the edge of the culture well (directly beneath the mesh) (A-F) and the middle of the well (G-L) after 24 h of direct exposure to the culture media (control, A and G), uncoated PP mesh (B and H), 22NS (C and I), 20-2NS(D and J), 22S(E and K), and 20-2S (F and L). Red dashed lines indicate the approximate position of the PP mesh. Calcein (green) and ethidium bromide (red) indicate cells boundaries and dead cell nucleus, respectively.

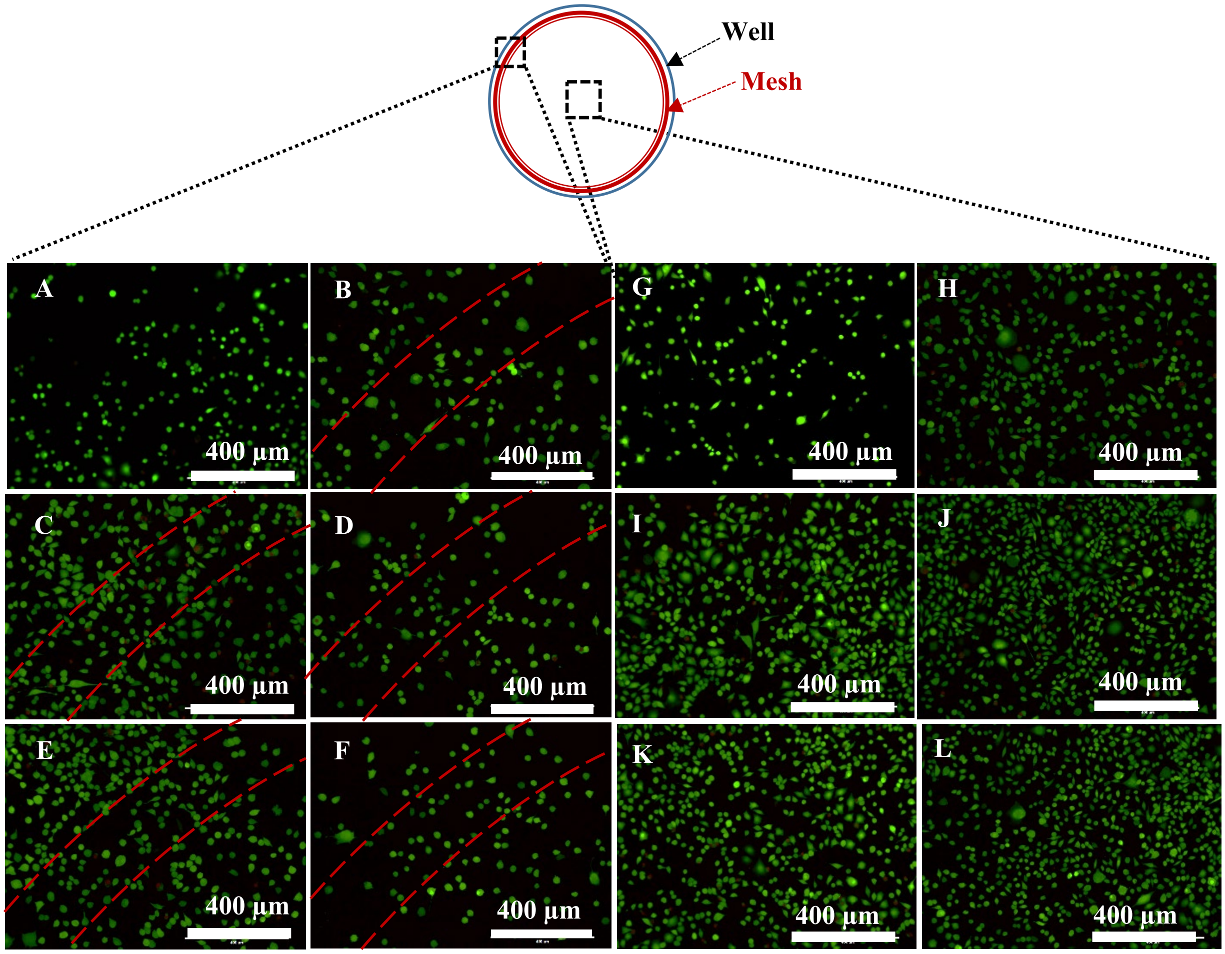

Figure S12. Images of live/dead staining of L929 fibroblast located at the edge of the culture well (directly beneath the mesh) (A-F) and the middle of the well (G-L) after 24 h of direct exposure to the culture media (control, A and G), uncoated PP mesh (B and H), 22NS (C and I), 20-2NS(D and J), 22S(E and K), and 20-2S (F and L) in the presence of 20-50 U/ml of catalase. Red dashed lines indicate the approximate position of the PP mesh. Calcein (green) and ethidium bromide (red) indicate cells boundaries and dead cell nucleus, respectively.

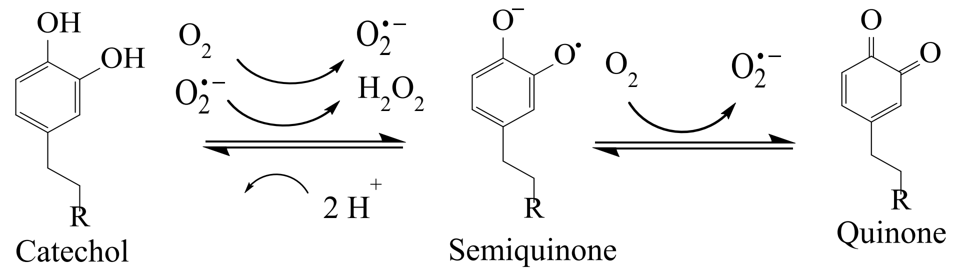

Figure S13. Proposed mechanism of catechol oxidation and hydrogen peroxide generation (H_2O_2).
